# Odour-mediated Interactions Between an Apex Reptilian Predator and its Mammalian Prey

**DOI:** 10.1007/s10886-022-01350-w

**Published:** 2022-03-01

**Authors:** Christopher R. Dickman, Loren L. Fardell, Nicole Hills

**Affiliations:** Desert Ecology Research Group, School of Life and Environmental Sciences, The University of Sydney, Sydney, NSW 2006 Australia

**Keywords:** Arms-race, Burrows, Diet, Giving-up density, Small mammals, *Varanus*

## Abstract

An important but understudied modality for eavesdropping between predators and prey is olfaction, especially between non-mammalian vertebrate predators and their prey. Here we test three olfactory eavesdropping predictions involving an apex reptilian predator, the sand goanna *Varanus gouldii*, and several species of its small mammalian prey in arid central Australia: 1) small mammals will recognize and avoid the odour of *V. gouldii*; 2) *V. gouldii* will be attracted to the odour of small mammals, especially of species that maximize its energetic returns; and 3) small mammals will be less mobile and will show higher burrow fidelity where *V. gouldii* is absent compared with where it is present. As expected, we found that small mammals recognized and avoided faecal odour of this goanna, feeding less intensively at food patches where the odour of *V. gouldii* was present than at patches with no odour or a pungency control odour. *Varanus gouldii* also was attracted to the odour of small mammals in artificial burrows and dug more frequently at burrows containing the odour of species that were energetically profitable than at those of species likely to yield diminishing returns. Our third prediction received mixed support. Rates of movement of three species of small mammals were no different where *V. gouldii* was present or absent, but burrow fidelity in two of these species increased as expected where *V. gouldii* had been removed. We conclude that olfaction plays a key role in the dynamic interaction between *V. gouldii* and its mammalian prey, with the interactants using olfaction to balance their respective costs of foraging and reducing predation risk. We speculate that the risk of predation from this apex reptilian predator drives the highly unusual burrow-shifting behaviour that characterizes many of Australia's small desert mammals.

**Supplementary Information:**

The online version contains supplementary material available at 10.1007/s10886-022-01350-w.

## Introduction

All organisms release chemical compounds into the environment. Some of these compounds are shed as excreta or produced during the decomposition process after death, whereas others are metabolic products that can convey specific information to conspecific or heterospecific individuals (Vos et al. 2006). Such infochemicals often affect interactions between organisms and thus may be profoundly important in influencing individual fitness, population dynamics, and the structure of ecological webs in terrestrial and aquatic ecosystems (Dicke and Sabelis 1988; Vos et al. 2006; Parsons et al. 2018; Müller et al. 2020). In fur seals for example, chemical fingerprints help to determine mother–offspring similarity and colony membership (Stoffel et al. 2015), and in many other taxa infochemicals shape preferences for particular mates or ensure recognition and avoidance of individuals from species that are otherwise ecologically similar (Caspers et al. 2009). Infochemicals also shape the outcomes of competitive, mutualistic, commensal, predatory, and parasitic interactions among species (e.g., Lewis et al. 2013; Allen et al. 2017; Chrétien et al. 2021; Scogings et al. 2021), and are being used increasingly in programs of conservation management (Norbury et al. 2021).

Olfactory interactions between prey and predators are prevalent and can have particularly important effects across two or more trophic levels (Jones et al. 2016). For prey organisms, selective benefits should accrue to individuals that recognize and respond appropriately to the olfactory cues of their predators. Among vertebrates, for example, prey may respond to a predatory odour cue by investigating, freezing, or fleeing from the site of the cue, and show acute or chronic stress (Fardell et al. 2020) as well as latent effects on their levels of vigilance behaviour, habitat use, temporal and spatial activity (Fenn and Macdonald 1995; Parsons and Blumstein 2010a, b; Cox et al. 2012; Parsons et al. 2018). These responses are often calibrated according to the specific identity of the predator and the level of threat that it poses to prey (Dickman 1992; Anson and Dickman 2013; Grau et al. 2019), and to factors such as the source of the odour (e.g. urine or faeces), its age and intensity (Fendt et al. 2020), and the prior experience of prey individuals with predators (Bleicher et al. 2018). In mammals olfactory information is processed by the amygdala and hypothalamus, allowing for learned behavioural responses of prey to predators (Takahashi 2014; Canteras et al. 2015). Intriguingly, prey may respond positively to the odour of dominant, or apex, predators if those predators actively suppress subordinate predators that pose greater risks to the prey; here the apex predator provides net benefit to the prey if its per capita predatory effect on prey is less than that of the subordinate predators (Jones et al. 2016). In situations where prey are naïve to predators and do not recognize their olfactory cues, such as where prey or predator species have been introduced to a new environment, prey mortality may be high and local populations will be at high risk of extinction (Salo et al. 2007).

For olfactory predators, by contrast, individuals should benefit if they can efficiently identify and locate prey by their odour (Hughes et al. 2010). If prey organisms are buried, sessile, or cryptically camouflaged (e.g., eggs in birds' nests), olfaction may be the only sensory modality by which predators can detect them (Conover 2007). For example, common shrews *Sorex araneus* use odour to locate fly pupae buried at depths up to 16 cm (Churchfield 1980), as do desert rodents when excavating buried seeds (Taraborelli et al. 2009) and grey squirrels *Sciurus carolinensis* selecting early-germinating scatter-hoarded seeds in preference to dormant seeds (Sundaram et al. 2020). If prey organisms are mobile, predators are more likely to hunt them by using odour cues deposited by prey in the environment or at focal sites such as burrows, nests or shelter sites. For example, wolves *Canis lupus* and other predators such as sharks can follow blood trails to locate prey (Tester 1963; Gable et al. 2016), while black rats *Rattus rattus* and other mammalian predators use odour to find bird nests (Price and Banks 2012; Ibáñez-Álamo et al. 2015). There is evidence that predators can detect and home in on prey by tracking the alarm odours that prey emit when fearful (Cocke and Thiessen 1986; Müller-Schwarze 2006), and emerging evidence that predators can distinguish the odours of prey that differ in physical condition, selecting those that are easiest and most energetically profitable to hunt (Newman and Buesching 2019). Reward-based learning allows predators to associate different prey odours with different levels of reward or energetic gain (MacArthur and Pianka 1966; Garvey et al. 2017). Using the vomeronasal system, predators also may exploit the ‘olfactory web’ and eavesdrop on the olfactory cues of other predators to reduce inter-species competitive interactions (Banks et al. 2016). Factors such as the age, strength, and patchiness of odour cues also affect detection success by predators (Carthey et al. 2011; Norbury et al. 2021).

Reptiles are seldomly featured as the top-down force in vertebrate predator–prey systems, rather they are commonly featured as prey items for larger mammalian or avian predators (e.g., Woinarski et al. 2018; Stobo-Wilson et al. 2021). However, there is compelling evidence that, in some contexts, reptilian predators represent a 'forgotten majority' (Sutherland and Bryant 2014), greatly influencing other vertebrates via competitive and predatory interactions (Savidge 1987; Sutherland et al. 2011; Radford et al. 2020), or as ecosystem engineers (Doody et al. 2020). Because of the selective advantages to prey of reducing their risk of detection by predators, and to predators of overcoming prey defences, arms-races can be expected that progressively modify the processes and outcomes of predator–prey dynamics (Banks et al. 2014). In a recent review, Newman and Buesching (2019) considered the arms-race between eavesdropping predators and their prey, and how conspicuous prey odours are deployed and spied upon, to be understudied but important areas in research in vertebrate systems. There is a particular dearth of information on how reptilian predators and their prey use infochemicals, although some snakes detect and track prey odours (e.g., Burghardt and Denny 1983; Webb and Shine 1992), as do some *Varanus* spp. (Pianka and Vitt 2003), We address these knowledge gaps here by exploring the role of olfaction in the interaction between an apex reptilian predator, the Australian sand goanna *Varanus gouldii* (Varanidae: 1–2 kg), and its mammalian prey. We examine whether these interactants recognize and respond to each others' odours, and also ask how the prey species minimize their risk of being detected and eaten by this goanna.

We selected this predator–prey system for several reasons. First, *V. gouldii* appears able to exploit prey odours (Garrett and Card 1993; Garrett et al. 1996), using its forked tongue to detect prey infochemicals before digging prey such as insects or small mammals from their burrows or under leaf litter (Pianka 1970, 1994; Thompson 1995). Prey is likely to be hunted once detected provided that it returns a net energetic gain (Losos and Greene 1988; Kaufman et al. 1996), with individuals rapidly learning new foraging techniques to reach detectable but not freely available foods (Cooper et al. 2019). In one study *V. gouldii* focussed its activity around the nests of a large (200–300 g) rodent, the greater stick-nest rat *Leporillus conditor*, apparently, because the smell of the rats' urine signalled a potential food source (Bolton and Moseby 2004). Second, in arid Australia small mammals use burrows to escape the climatic extremes, potentially leaving residual odours at burrow entrances that could be exploited by *V. gouldii*. Rodents construct 25–100 cm deep burrows, whereas co-occurring small dasyurid marsupials use soil cracks or usurp the burrows of larger animals (Dickman 1993, 2003). Many of these species are highly mobile and frequently move burrow locations (Letnic 2002; Haythornthwaite and Dickman 2006), resulting in home ranges that are unstable or nomadic. Baker and Dickman (2018) speculated that such nomadism—which is highly unusual and perhaps globally unique in small desert mammals—reduces the accumulation of prey odours at burrows and hence the risk of being detected and depredated by *V. gouldii*, although this hypothesis remains to be tested.

We tested the following predictions:

1. Small mammals will recognize and avoid the odour of *V. gouldii*,
2. *Varanus gouldii* i) will be attracted to the odour of small mammals, and ii) will be more attracted to the odours of species that maximize its energetic return, and
3. Small mammals will be less mobile and will show higher burrow fidelity where *V. gouldii* is absent compared with where it is present.

The main small mammals in our study system were the spinifex hopping-mouse *Notomys alexis* (~ 30 g), long-haired rat *Rattus villosissimus* (~ 120 g), sandy inland mouse *Pseudomys hermannsburgensis* (~ 12 g) and lesser hairy-footed dunnart *Sminthopsis youngsoni* (~ 10 g). The first two species dig deep (1 m) multi-entrance burrows that would be energetically costly for *V. gouldii* to excavate. By contrast, the latter two species occupy burrows that are simple and usually < 30 cm deep, and hence likely to be more profitable for *V. gouldii* to investigate and excavate. We used faecal analysis to confirm that these species occur in the diet of *V. gouldii* (Online Resource 1).

## Methods and Materials

### Study Area

Fieldwork was carried out at three sites on Ethabuka Reserve, a 215,000 ha area dominated by long red sand dunes on the north-eastern edge of the Simpson Desert, Queensland, Australia. The sites were Main Camp (23°46' S, 138°28' E), Field River (23°48' S, 138°04' E) and Way Site (23°47' S, 138°22' E), all of which contained populations of the study species ((Downey and Dickman 1993; Dickman et al. 2014). The dominant vegetation of the dune field is hard spinifex (*Triodia basedowii*) with stands of gidgee trees (*Acacia georginae*) on harder soil in the valleys between dunes (Wardle et al. 2015). Daily temperatures in summer exceed 40 °C and fall below 5 °C overnight in winter, and annual rainfall averages < 200 mm (Greenville et al. 2012). Wildfires may occur 1–2 years after heavy rains and have a mean minimum return interval of 26–27 years (Greenville et al. 2009). Heavy rain (552 mm) fell in 2010 near the beginning of the present study and was followed by wildfires in 2011 (Verhoeven et al. 2020). Our experiments were carried out in unburnt vegetation.

### Experiments

## Prediction 1: Small Mammals will Recognize and Avoid the Odour of *V. gouldii*

To test our first prediction, we used faecal material from *V. gouldii* as an odour source to present to small mammals. *Varanus gouldii* was captured during a long-term live trapping program in the study area (Dickman et al. 1999a, 2010, 2014; Greenville et al. 2016a) or by hand noosing following opportunistic sightings, and faeces produced during handling were placed in vials and frozen at -2 – -4 °C within 30 min. We used a giving-up density (GUD) experiment to gauge small mammal responses to the odour. The GUD is the amount of food that remains when an animal has finished foraging in a patch with an enriched food source. We provided food in an inedible matrix and allowed animals to choose between treatments with these food sources. If there are no constraints on foraging, individuals should spend adequate time in patches and consume as much food as needed. However, if an animal experiences a stressor, such as predation risk, in a food patch then GUDs increase, and animals spend less time in the patch (Brown 1988; Kotler et al. 1991).

To implement a GUD experiment, we established 54 sites, each > 25 m apart, along the bases or mid-sections of sand dunes where *V. gouldii* and small mammals were active. At each site we set up a food patch. This was a half-buried plastic bowl (15 cm diameter, 4 cm deep) containing food of high value to small mammals: mealworms *Tenebrio molitor*, which were energetically profitable for *S. youngsoni* (Fisher and Dickman 1993) or peanut quarters, which we considered would be of value to the study rodents (Murray and Dickman 1994; Murray et al. 1999). Either 10 mealworms or 10 peanut quarters were placed in each bowl, mixed into 200 ml of sifted sand. The underside of the bowls was smeared with a mixture of Vaseline and Coopex insecticide powder (Bayer, Pymble, New South Wales) to repel ants from the bowls. Twenty-seven bowls were set with each type of food, with the order of placement randomized. To assess small mammal responses to *V. gouldii* odour, we used cotton buds—6 cm-long sticks tipped with absorbent cotton wool (Johnson & Johnson, Sydney, New South Wales)—for odour presentation. The tip of a cotton bud was applied to fresh goanna faeces and then the stick was inserted vertically into the centre of a food bowl such that the odour-bearing cotton tip was ~ 2 cm above the bowl surface. To quantify the magnitude of response to *V. gouldii* odour, we also used cotton buds dipped in eucalyptus oil as a pungency control treatment (i.e., representing a strong but familiar odour) and cotton buds dipped in water as a procedural control (Kovacs et al. 2012). The three treatment odours were allocated randomly to bowls with each food type, so that n = 9 for each odour for the bowls containing mealworms and n = 9 for bowls with peanut quarters.

As our target small mammal species are nocturnal, the bowls were set up in the early evening and revisited near dawn when foraging was expected to have concluded. The GUD (number of mealworms or peanuts remaining in each bowl) was then recorded. The bowls were set with food but without the odour treatment for one night to allow animals to accustom to them. The bowls were then recharged each evening with fresh food (mealworms and peanut quarters) and new cotton buds with fresh odours (sand goanna, pungency, procedural control) over seven consecutive nights. Disposable latex gloves were used at all times to minimize cross-contamination of odours. To identify the small mammal species that had visited the food bowls, and thus whose GUDs were being measured, we smoothed the sand in a 10 cm-radius around each bowl and examined the footprints that were left each morning. The prints of the target species are readily distinguishable by differences in size, numbers of toes and imprint of the heel (Moseby et al. 2009; Dickman et al. 2010). GUDs could be recorded reliably if a bowl had been visited by one species or, if two or more species had visited, we could clearly discern the last forager by the overprinting of its tracks on those of earlier foragers. We discarded the results for any bowls if we could not reliably read the prints or if the site had been disturbed by wind or other species such as Australian ravens *Corvus coronoides*. The experiment testing our first prediction was carried out once, at Main Camp, in November 2014.

## Prediction 2: *Varanus gouldii* will be Attracted to the Odour of Small Mammals

To test our second prediction, we created artificial burrows that simulated those of the study mammals. The burrows were made by hammering PVC pipe into the soil and then gently removing it, with soil inside, to create a vertical 'burrow' that was 2.5 cm or 3.5 cm wide and ~ 25 cm deep. The narrower (2.5 cm) burrows were used on one occasion only (September 2012) and then abandoned in favour of the wider burrows to facilitate ease of creation and subsequent manipulation of odours. Clusters of four burrows were constructed at 32 – 50 sites along the bases or mid-sections of sand dunes, with each cluster spaced > 200 m from the next. In sand dune habitat *V. gouldii* occupies activity areas of 5.9 ha (274 m linear distance if the area is assumed to be circular), but take a week to cover them (Bolton and Moseby 2004). Thus we assumed that our burrow clusters would be accessed by no more than a single *V. gouldii* provided they were set for a week or less. By contrast, the artificial burrows within a site were spaced 2 – 3 m apart so that an individual *V. gouldii* encountering one burrow in a cluster would have an approximately equal chance of encountering the others.

We tested the ability of *V. gouldii* to find and discriminate prey odours by placing small balls of cotton wool bearing the body odour of the study species at the bottom of each artificial burrow. Body odours were used in preference to urinary or faecal odours as integumentary chemicals are most likely to be deposited in burrows as small mammals enter and exit them. Within each cluster, we presented the odour of three species of small mammals—one odour per artificial burrow—and placed fresh cotton wool in the fourth burrow to serve as an odourless control. Field visits in September and November 2012 used the odours of *N. alexis*, *P. hermannsburgensis,* and *R. villosissimus*; the latter species disappeared from the study area in 2012 (Greenville et al. 2013), and its odours were replaced by those of *S. youngsoni* for experiments carried out in September 2013, April and November 2014. We term these two periods *phase 1* and *phase 2*, respectively. Cotton wool was imbued with the odour of these species by providing captive or wild-caught animals with balls of this material as bedding for periods of > 12 h. The material was then frozen at -2 – -4 °C to reduce degradation of volatile components (Fardell et al. 2021), and inserted into the artificial burrows by operators wearing latex gloves and using clean forceps. As the cotton wool could be seen at the bottom of the burrows when the sun was overhead, a small (4 × 4 cm) patch of clean brown cloth was placed above the cotton wool to obscure it and ensure that odour was the only cue provided to foraging *V. gouldii*. Sand in a 30 cm radius around each artificial burrow was smoothed to capture the tracks of visiting *V. gouldii*.

The burrows and their experimental odours were set up in the mornings before *V. gouldii* became active, and were checked in the evening. We recorded the tracks of *V. gouldii* in the 30 cm radius around burrows and whether burrows had been dug into. This goanna characteristically excavates burrows by tearing at the soil from one side of a burrow with their forelimbs and following the burrow to the bottom where prey is usually located. Here, we recorded digging if a burrow had been partially or fully excavated. If a burrow in a cluster had been dug, we relocated the burrow cluster to a new position about 10 m away. All burrow sites were moved to new positions after 2 – 7 days; varanids quickly learn to distinguish profitable from non-profitable feeding sites and modes of prey capture (Manrod et al. 2008), and we assumed that individual *V. gouldii* would lose interest in small mammal odours with successive unsuccessful digs. Cotton wool balls bearing the experimental odours were replenished every 2 days to maintain their freshness, and inspections of goanna activity were made each evening.

## Prediction 3: Small Mammals will be less Mobile and will Show Higher Burrow Fidelity in the Absence than the Presence of *V. gouldii*

Test of our third prediction required a small scale experimental removal and relocation of *V. gouldii* so that we could compare small mammal mobility and burrow fidelity in the presence and absence of this apex reptilian predator. We selected an area of ~ 50 ha at Main Camp with access tracks and three trapping grids on which we could capture *V. gouldii*, and commenced a removal program in September 2009. Between this time and November 2011, 39 *V. gouldii* were captured and relocated in similar habitat > 5 km away. This goanna is not territorial and occupies areas that shift over time as animals track prey (Bolton and Moseby 2004), and our tracking of a subset (n = 11) of relocated individuals indicated that they rapidly established new burrows and maintained their mass and condition following translocation (CD, NH, unpub. data).

To gauge the degree to which the removal area was free of *V. gouldii*, we set up three 30 m × 1 m transects on each of the three removal grids and a further nine transects on unsealed vehicle tracks through the removal area. The transects were raked and then smoothed by dragging a half-filled hessian sack along their length to create a suitable surface to record animal tracks, and were checked daily for three days on each of six field trips between September 2009 and November 2011. The tracks of *V. gouldii* were recorded on 12 of the 18 transects (67%) in September 2009 but fell to 0 on three field trips in 2011 as the removal protocol progressed. It is likely that some *V. gouldii* activity remained in the removal area in 2011 as we found tracks away from the transects on two occasions in that year. However, the results indicate that activity of *V. gouldii* on the removal area had been reduced to nearly zero. By contrast, similar monitoring at the same time of a control area 7 km away, where no removals were undertaken, showed that *V. gouldii* activity on transects remained relatively unchanged. There, 10 of 18 transects (56%) yielded sand goanna tracks in September 2009, compared with 8 – 14 of the 18 transects (44% – 78%) on the field trips in 2011.

In view of the above results, we used radio-tracking to quantify small mammal mobility and burrow fidelity in the control and removal areas on three occasions in May, August, and October – November 2011. Small mammals were live-trapped on long-term trapping grids in each area (Dickman et al. 1999b, 2014) and equipped with single-stage radio-tags with 10-cm trailing whip antennae that were attached either using a plastic cable tie collar (*N. alexis* and *P. hermannsburgensis*) or by cyanoacrylate glue to fur between the scapulae (*S. youngsoni*); no *R. villosissimus* were radio-tracked. Tags weighed 0.4 – 1.0 g and were attached to animals that were randomly selected from those captured provided that their weight gain with the tag was < 5%. Tags were supplied by Biotrack (Wareham, United Kingdom), Holohil (Ontario, Canada), or Titley Electronics (Ballina, New South Wales, Australia) and used frequencies between 150 and 151 MHz. Detailed protocols for tag fitting and animal release are provided by Dickman et al. (2010) and Haythornthwaite and Dickman (2006).

Animals were located at night every 1–4 h using a 3-element hand-held Yagi antenna and a Regal 1000 (Titley Electronics, Ballina, New South Wales, Australia) or TR-2 (Telonics Inc., Mesa, Arizona) receiver. If animals were in open areas, we approached them from downwind, walking on sand to reduce noise and using red light to establish visual contact to determine their location precisely. If animals were in covered areas or could not be approached closely, we estimated locations by triangulation of 2–3 bearings taken in the direction of the peak signal using a prismatic compass. Bearings were taken from known triangulation points ensuring that angles were > 20° and < 120° from each other (Kenward 2001). Pilot trials using tags placed at known locations indicated that bearings were accurate to within ± 5° up to distances of ~ 110 m (Dickman et al. 2010). By day, we located animals in burrows by walking along dune crests to pick up their signals and then walking along the line of peak signal strength. Burrows were pinpointed by removing the Yagi antenna and homing in on the signal, to within 1–2 m of the animal, using the coaxial cable and receiver. Animal locations and bearings were flagged using marker tape and later placed on fine-scale maps of the study area.

Although all components of this research were approved by the University of Sydney Animal Care and Ethics Committee, we took particular care with animal handling in the tests of our third prediction. No animals were used in radio-tracking if they appeared distressed upon capture and handling, and were instead released at the point of capture. Animals that were recaptured at the conclusion of radio-tracking had their tags removed; none had lost more than 4% of their body mass and all appeared to be in good condition.

### Data Processing and Statistical Analyses

To test our first prediction concerning recognition and avoidance by small mammals of *V. gouldii* odour, we compared GUDs between the three odour treatments (no odour – *control*; pungency control – *eucalyptus*; goanna faecal material – *goanna*) at all sites that were visited, using a linear mixed-effects model. Treatment and small mammal species were set as fixed effects and survey day as a random effect in a *lmer* model using the *lme4* package (Bates et al. 2015) in *R*. Stepwise comparisons indicated that food type and GUD station had little effect on model results, and thus were not retained in the most parsimonious model. Model fit was assessed using residual diagnostics (Hartig 2021). Post hoc comparisons between the treatment groups and for each species between treatments were made on the final model using estimated marginal means via *emmeans* (Lenth 2021), which allows for the error variances that have been specified in the model, and Tukey *P*-value adjustments for comparing the families of three and twelve estimates.

To test our second prediction concerning the attraction of *V. gouldii* to small mammal odour, we counted the numbers of artificial burrows that elicited a response from *V. gouldii*. Responses were recorded as digging activity or as tracks within 30 cm of the artificial burrows that indicated investigative activity, and were analyzed separately. Analyses were carried out separately for the species complements present in 2012 (i.e., with the inclusion of *R. villosissimus* – phase 1) and 2013–2014 (with *S. youngsoni* – phase 2). Survey session was considered a nominal factor that differed for each new location of the artificial burrows within one of the three main study locations. Survey day within each survey session was also considered a nominal factor. Generalized least squares (GLS) models were used to account for heterogeneity of variances, which were observed across the treatments, survey days, survey sessions, and locations. Following Zuur et al. (2009) we incorporated the nominal variables using the *nlme* package (Pinheiro et al. 2021) in *R* to implement GLS with a variance structure that allowed for differences in variance across survey days per survey session per location. We also incorporated an exponential variance structure for the covariance to account for the variance spread across different survey sessions alone or by survey day per survey session, depending on the data and best fit model. Log likelihood and Akaike information criterion comparisons were used to determine the most parsimonious models. The fixed explanatory variables retained in all models were treatment odour, survey day, and survey session; location was not retained as it had no significant effect. The only other difference between the models was that survey day was not retained in the exponential variance structure for models of the track data for both species complements*.* Graphical validation of the optimal model was obtained by comparing box plots of the raw data against the model estimates, by plotting the residuals against the fitted values for the nominal explanatory variables, and via histograms and Q-Q plots of the model residuals (Zuur et al. 2009). Post hoc comparisons between the treatment groups were made on the final model using estimated marginal means via *emmeans* (Lenth 2021), which allows for the error variances that have been specified in the GLS model, and Tukey *P*-value adjustments for comparing a family of four estimates. All analyses were carried out in *R* (R core team 2021).

To analyse small mammal movements and burrow fidelity (prediction 3), we mapped the locations of animals that we had sighted and estimated the positions of triangulated animals using an iterative maximum likelihood estimator (LOAS, Ecological Software Solutions, Sacramento, California). Movements were calculated as the distance covered between successive signal locations, per unit time (m/h), with the distance (*d*_*i*_) moved by an individual between its first signal location *i*(*x*_*i*_, *y*_*i*_) and the next (*x*_*i*+1_, *y*_*i*+1_) calculated using White and Garrott’s (1990) equation:

$$di=\surd {\left({x}_{i}+1 - {x}_{i}\right)}^{2}+{({y}_{i}+1-{y}_{i})}^{2}$$

We assessed small mammal fidelity to burrows, (*f*), using the index: *f* = *N*_max_/*N.*

where *N*_max_ is the maximum number of visits by an individual to the same burrow over the period of radio-tracking, and *N* is the total number of its visits to all burrows. Index values thus range from *f* = 1 for an individual that uses a single burrow to *f* = 1/*N* for an individual that uses all burrows once. The index formula is the same as the Berger-Parker index of species dominance (Magurran 2004), and its use here follows Dickman et al. (2010). Only small numbers of the study species were radio-tracked on each of the three sampling occasions, so results were pooled and rates of movement and burrow fidelity compared for each species between the control and *V. gouldii* removal areas using analysis of variance. The burrow fidelity data for *S. youngsoni* were log-transformed to improve the variance structure prior to analysis; no other transformations were required.

## Results

### Prediction 1: Small Mammals will Recognize and Avoid the Odour of *V. gouldii*

On average, more than half the GUD sites (57%) were visited each night by the study species, with another 11% of sites disturbed by wind or other species. *Rattus villosissimus* was absent from the study area in November 2014 and hence provided no results. However, small numbers of another *Sminthopsis* species, the hairy-footed dunnart *S. hirtipes* (15 g), were present, allowing its response to *V. gouldii* odour to be investigated. More visits were made by the study species to GUD sites with no odour over the seven nights of the experiment (total visits = 91: *N. alexis*, n = 17; *P. hermannsburgensis*, n = 31; *S. youngsoni*, n = 39; *S. hirtipes*, n = 4) than to sites with the pungency control (total visits = 80: *N. alexis*, n = 16; *P. hermannsburgensis*, n = 28; *S. youngsoni*, n = 32; *S. hirtipes*, n = 4), with fewest visits made to sites with the odour of *V. gouldii* (total visits = 43: *N. alexis*, n = 9; *P. hermannsburgensis*, n = 14; *S. youngsoni*, n = 18; *S. hirtipes*, n = 2).

**Fig. 1:**
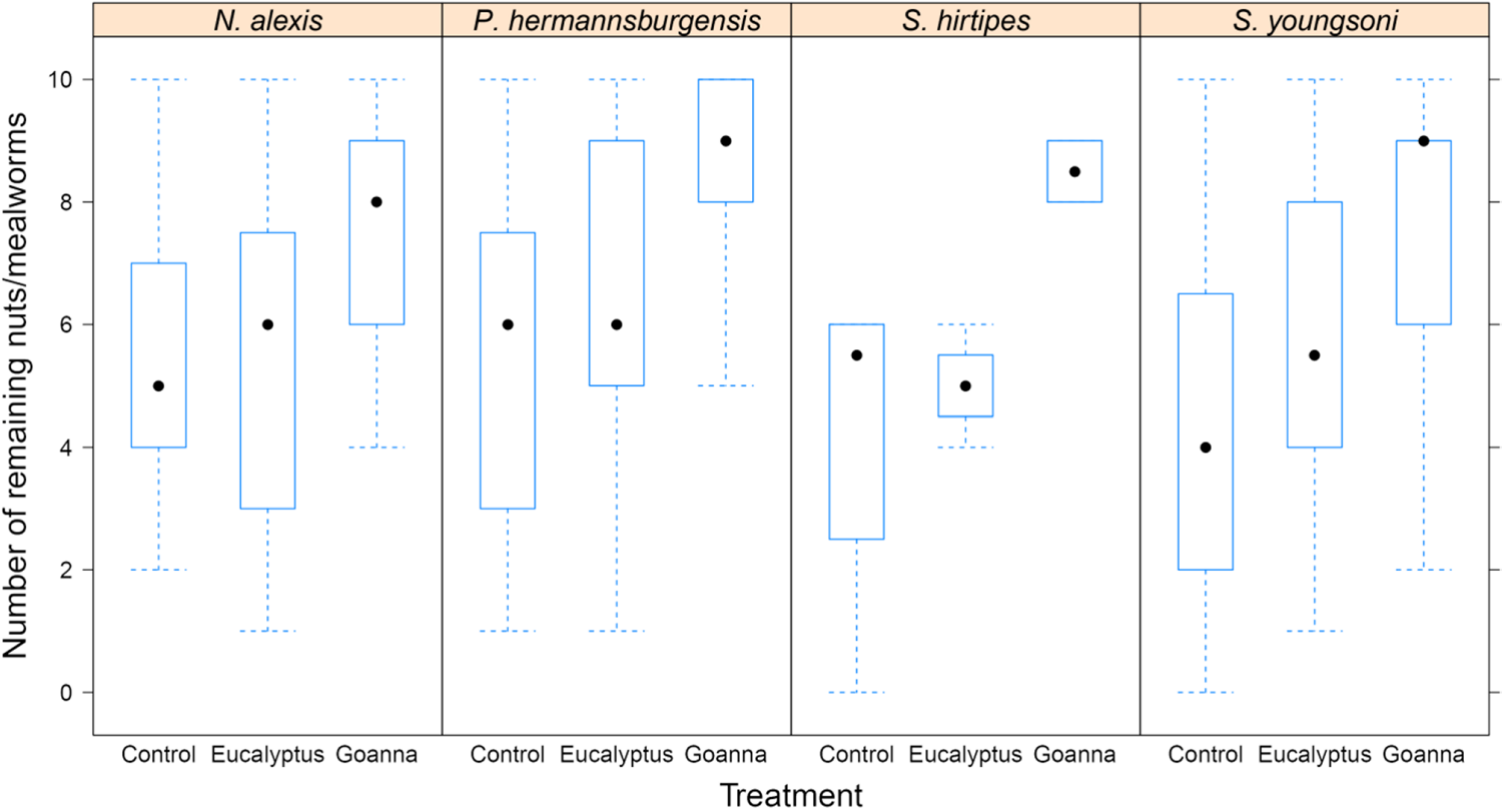
Boxplots of raw data showing the giving-up density (GUD) responses of four species of small mammals to treatments of no odour (*Control*), a pungency control (*Eucalyptus*), and sand goanna *Varanus gouldii* faecal material (*Goanna*) at food patches. The y-axis shows the number of food items, mealworms or peanuts, left behind at food patches after overnight periods of foraging (the GUD)

The foraging responses of all four species were similar across the three odour treatments. GUDs were significantly higher (more food remained after foraging) in the treatment bearing the odour of *V. gouldii* than in the control and pungency control treatments (Table 1, Fig. 1).

**Table 1:** Linear mixed-effects model results of small mammal giving-up density (GUD) responses to treatments of no odour (*Control*), a pungency control (*Eucalyptus*), and sand goanna *Varanus gouldii* faecal material (*Goanna*) at food patches. Estimates are given as the first treatment in the contrast as compared to the second, larger estimated mean. Significant results are denoted by an asterisk

Contrast	Estimate	SE	Df	*t*	*P*
Control—Eucalyptus	-0.9540	0.3960	203.000	-2.4090	0.0443*
Control—Goanna	-3.1080	0.4790	204.000	-6.4860	< 0.0001*
Eucalyptus—Goanna	-2.1540	0.4890	203.000	-4.4070	0.0001*
*Notomys alexis*: Control—Goanna	-3.1077	0.4790	204.000	-6.4860	< 0.0001*
*Notomys alexis*: Eucalyptus—Goanna	-2.1538	0.4890	203.000	-4.4070	0.0010*
*Pseudomys hermannsburgensis*: Control—Goanna	-3.1077	0.4790	204.000	-6.4860	< 0.0001*
*Pseudomys hermannsburgensis*: Eucalyptus—Goanna	-2.1538	0.4890	203.000	-4.4070	0.0010*
*Sminthopsis hirtipes*: Control—Goanna	-3.1077	0.4790	204.000	-6.4860	< 0.0001*
*Sminthopsis hirtipes*: Eucalyptus—Goanna	-2.1538	0.4890	203.000	-4.4070	0.0010*
*Sminthopsis youngsoni*: Control—Goanna	-3.1077	0.4790	204.000	-6.4860	< 0.0001*
*Sminthopsis youngsoni*: Eucalyptus—Goanna	-2.1538	0.4890	203.000	-4.4070	0.0010*

### Prediction 2: *Varanus gouldii* will be Attracted to the Odour of Small Mammals

**Fig. 2:**
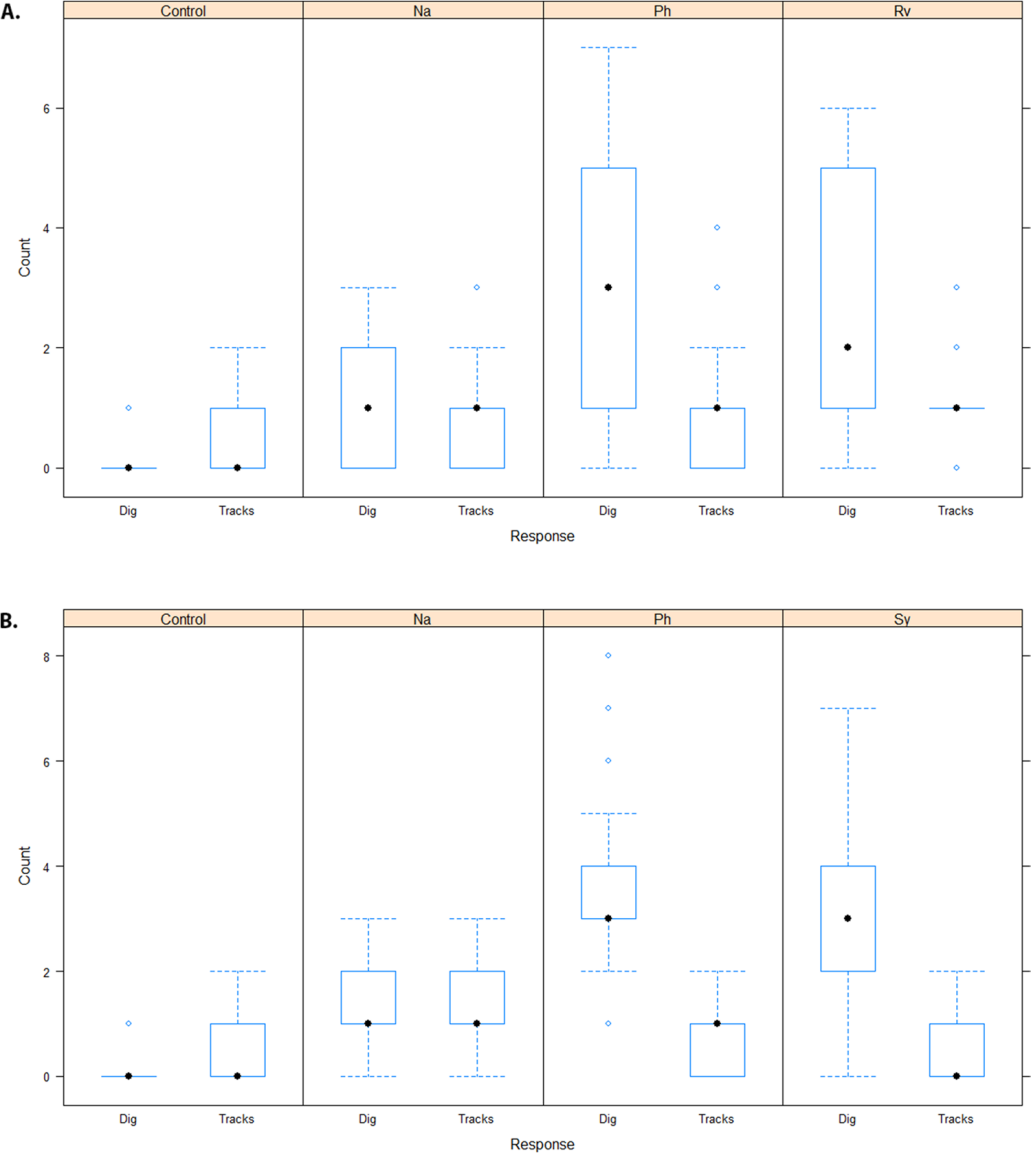
Boxplots of raw data showing the digging (dig) and investigative (tracks) responses of sand goannas *Varanus gouldii* at artificial burrows that contained no odour (control) and the odours of small mammalian prey species in two time periods, Phase 1: A) *Notomys alexis*, *Pseudomys hermannsburgensis*, and *Rattus villosissimus*; and Phase 2: B) *Notomys alexis*, *Pseudomys hermannsburgensis*, and *Sminthopsis youngsoni*. The y-axis shows the count of artificial burrows that were observed to have been dug up or visited each day

In phase 1, when *R. villosissimus* was present, *V. gouldii* dug up or scratched at the entrance of artificial burrows containing the odour of *R. villosissimus* on 80 occasions over 1365 'burrow-days' (1 burrow-day = 1 burrow containing the species' odour over 1 day), and in phase 2 V*. gouldii* showed digging activity at burrows containing the odour of *S. youngsoni* on 88 occasions over 2028 burrow-days. Over both phases *V. gouldii* showed digging activity at burrows containing the odour of *N. alexis* on 79 occasions and at burrows with *P. hermannsburgensis* odour on 203 occasions over a total of 3393 burrow-days. Control burrows were excavated or scratched at on only seven occasions over 3393 burrow-days. Tracks of *V. gouldii*, with no digging activity, followed a similar pattern but were recorded less frequently (Fig. 2).

Model results showed that *V. gouldii* excavated burrows containing small mammal odour significantly more often than the odourless control burrows in both phase 1 and phase 2 (Table 2, Fig. 2). Digs at *P. hermannsburgensis* burrows occurred consistently in both phases (Table 2, Fig. 2), but were not significantly more frequent than those at the burrows of *R. villosissimus* in phase 1 or *S. youngsonii* in phase 2 (Table 3). However, *V. gouldii* dug at burrows containing the odour of *N. alexis* significantly less often than at those containing the odours of other small mammals (Table 3). The investigative response of *V. gouldii*, as shown by tracks, was also less at control, odour-free burrows than at all other treatment burrows except for those containing the odour of *S. youngsoni*; burrows with the odour of this latter species elicited very little response from *V. gouldii* (Table 2, Fig. 2).

**Table 2:** Generalised least squares model results of digging and investigative (track) responses of sand goannas *Varanus gouldii* at artificial burrows that contained no odour (*Control*) or the odours of small mammalian prey species in two time periods: Phase 1, *Notomys alexis* (Na), *Pseudomys hermannsburgensis* (Ph), and *Rattus villosissimus* (Rv); and Phase 2, *Notomys alexis* (Na), *Pseudomys hermannsburgensis* (Ph), and *Sminthopsis youngsoni* (Sy). Significant results are denoted by an asterisk

Response	Treatment	Estimate	SE	*t*	*P*
Phase 1					
Digging	Control	0.7537	0.3628	2.0772	0.0403*
Digging	Na	0.8801	0.3048	2.8877	0.0047*
Digging	Ph	2.2072	0.3048	7.2419	< 0.0001*
Digging	Rv	1.9013	0.3048	6.2385	< 0.0001*
Tracks	Control	0.0610	0.1869	0.3264	0.7448
Tracks	Na	0.6258	0.1709	3.6611	0.0004*
Tracks	Ph	0.3406	0.1709	1.9926	0.0490*
Tracks	Rv	0.5556	0.1709	3.2503	0.0016*
Phase 2					
Digging	Control	-0.5076	0.4245	-1.1956	0.2345
Digging	Na	1.2971	0.3172	4.0887	0.0001*
Digging	Ph	3.5809	0.3172	11.2881	< 0.0001*
Digging	Sy	2.8333	0.3172	8.9313	< 0.0001*
Tracks	Control	0.4541	0.2360	1.9245	0.0570
Tracks	Na	0.6910	0.1824	3.7881	0.0003*
Tracks	Ph	0.3638	0.1824	1.9944	0.0487*
Tracks	Sy	-0.0359	0.1824	-0.1968	0.8444

**Table 3:** Estimated marginal means in treatment group comparisons from generalised least squares models testing digging and investigative (track) responses of sand goannas *Varanus gouldii* at artificial burrows that contained the odours of small mammalian prey species in two time periods: Phase 1: *Notomys alexis* (Na), *Pseudomys hermannsburgensis* (Ph), and *Rattus villosissimus* (Rv); and Phase 2: *Notomys alexis* (Na), *Pseudomys hermannsburgensis* (Ph), and *Sminthopsis youngsoni* (Sy). Significant results are denoted by an asterisk

Response	Comparison	Estimate	SE	Df	*t*	*P*
Phase 1						
Digging	Na—Ph	-1.327	0.305	60.5	-4.354	0.0003*
Digging	Na—Rv	-1.021	0.305	60.5	-3.351	0.0074*
Digging	Ph—Rv	0.306	0.305	60.5	1.003	0.748
Tracks	Na—Ph	0.2852	0.171	93.3	1.668	0.3461
Tracks	Na—Rv	0.0702	0.171	93.3	0.411	0.9765
Tracks	Ph—Rv	-0.215	0.171	93.3	-1.258	0.592
Phase 2						
Digging	Na—Ph	-2.284	0.317	106	-7.199	< 0.0001*
Digging	Na—Sy	-1.536	0.317	106	-4.843	< 0.0001*
Digging	Ph—Sy	0.748	0.317	106	2.357	0.092
Tracks	Na—Ph	0.3272	0.182	106	1.794	0.2821
Tracks	Na—Sy	0.7269	0.182	106	3.985	0.0007*
Tracks	Ph—Sy	0.3997	0.182	106	2.191	0.1324

### Prediction 3: Small Mammals will be less Mobile and will Show Higher Burrow Fidelity in the Absence than the Presence of *V. gouldii*

Between six and eight individuals of each small mammals species were radio-tracked over periods of 2–7 days, with 5–31 location fixes obtained per individual. Rates of movement (m/h: grand mean ± SE) varied between species (*N. alexis*: 87.8 ± 22.4; *P. hermannsburgensis*: 93.0 ± 22.2; *S. youngsoni*: 239.8 ± 39.1), but were no different for each species when compared between the control and *V. gouldii* removal areas (Table 4). Overall, animals used 42 different burrows on 78 occasions, with individuals of each species using either the same burrow or different burrows for 4–5 consecutive days. Burrow fidelity (grand mean ± SE) was relatively high for *N. alexis* (0.807 ± 0.077) and no different for this species between the control and *V. gouldii* removal areas (Table 4). Overall burrow fidelity was less for both *P. hermannsburgensis* (0.604 ± 0.078) and *S. youngsoni* (0.586 ± 0.140), with fidelity to burrows less in the control area than in the area where *V. gouldii* had been removed (Table 4).

**Table 4:** Rates of movement (m/h) and fidelity to the same burrow by three species of radio-tracked small mammals in areas where the sand goanna *Varanus gouldii* was present (+ Goanna) and absent (- Goanna); means are shown ± SE with the number of each species in each treatment that were tracked (n), and significant results are denoted by an asterisk

	Treatment		*F*	*P*
	+ Goanna	- Goanna		
*Notomys alexis*				
Rate of movement	71.0 ± 23.12 (n = 3)	104.67 ± 41.15 (n = 3)	0.509	0.515
Burrow fidelity	0.671 ± 0.089 (n = 3)	0.943 ± 0.057 (n = 3)	6.626	0.062
*Pseudomys hermannsburgensis*				
Rate of movement	66.75 ± 18.98 (n = 4)	119.25 ± 38.43 (n = 4)	1.510	0.266
Burrow fidelity	0.458 ± 0.058 (n = 4)	0.750 ± 0.102 (n = 4)	6.156	0.048*
*Sminthopsis youngsoni*				
Rate of movement	294.33 ± 43.35 (n = 3)	185.33 ± 53.01 (n = 3)	2.534	0.187
Burrow fidelity	0.303 ± 0.027 (n = 3)	0.867 ± 0.133 (n = 3)	28.30	0.006*

## Discussion

The results provide general support for our three initial predictions. Thus, small mammals that form part of the diet of *V. gouldii* recognised and avoided the odour of this apex reptilian predator, feeding less intensively at sites with *V. gouldii* odour than at sites with a pungency control or no odour. In its turn, *V. gouldii* was attracted to artificial burrows containing the odour of small mammals, and dug more frequently at burrows with small mammal odour than at odourless control burrows. Finally, two of three species of small mammal showed lower fidelity to their burrows in the presence than in the absence of *V. gouldii*, suggesting that this goanna exerts an influence on the tenure of small mammals in their burrow systems. These findings are the first to demonstrate reciprocal odour-based interactions between an apex reptilian predator and small mammalian prey, and provide novel evidence that top down predation by a varanid contributes to the nomadic burrow-shifting behaviour that distinguishes many of Australia's small desert mammals from their counterparts in other world deserts. We discuss the implications of our results in more detail below.

The GUD results provided a strong indication that small mammals recognize and avoid the odour of *V. gouldii*, and the similarity in GUD response by each species to the no-odour and pungency control treatments suggests further that this response is specific and not due to odour strength or volatility. The distribution of *V. gouldii* largely encompasses that of each of the small mammal species, with the area of overlap exceeding a million square kilometres in arid central Australia (cf. distribution maps in Cogger 2014 and Van Dyck and Strahan 2008). As the aridification of Australia began in the mid-Miocene some 15 million years ago and intensified over the last 1 – 4 million years (Byrne et al. 2008), it is therefore likely that coevolution between *V. gouldii* and its mammalian prey has been prolonged and extensive. Comparative research on the responses of mammals to other varanid predators is limited, but our findings accord with strong aversive responses that are shown by the common ringtail possum *Pseudocheirus peregrinus* to the faecal odour of the lace monitor *Varanus varius* (Anson and Dickman 2013).

Although odour cues such as predator faeces provide ostensibly well-founded information to prey about predation risk, prey responses are often variable and balanced according to the degree of risk that they perceive (Dickman 1992; Apfelbach et al. 2005; Sih et al. 2010) or to differences in past experience, age or health (Bedoya-Pérez et al. 2019). Our GUD results suggest that *V. gouldii* faecal odour conveys considerable risk to small mammals. This varanid includes small mammals in its diet and the presence of fresh faeces therefore should provide reliable information that a predator is not far away (Canteras et al. 2015). However, all the small mammals are nocturnal and *V*. *gouldii* is diurnal (Gordon et al. 2010), so it might be expected that small mammals could forage relatively safely even in places where odour of the varanid is strong. There are two reasons why such foraging may still be risky. Firstly, in the hot and often humid conditions that prevail over the Austral summer in arid Australia, some crepuscular activity of *V*. *gouldii* and small mammals is likely, thus increasing the chance of direct encounters between predator and prey. Secondly, if site-based foraging is prolonged—as would be needed to produce low GUDs—this would intensify the deposition of odour by small mammals at the foraging sites, and thus leave stronger cues to investigate for nearby *V*. *gouldii*. In both cases small mammals should reduce their risk of predation by minimising their foraging time where fresh *V. gouldii* faecal odour is present compared with where this odour cue is absent.

The results showed further that the four species of small mammals to visit GUD bowls responded similarly to the odour treatments. Given that *N. alexis* and *S. hirtipes* occurred less frequently than *P. hermannsburgensis* in the diet of *V. gouldii* (Online Resource 1), this might suggest that there are differences in predation risk among these species and hence also that species-specific differences in the magnitude of their GUD responses would be expected. This expectation is all the more reasonable if *V. gouldii* targets some species more intensively than others or if the small mammal species differ in their ability to escape once detected, as occurs in the interaction of small mammals with feral cats *Felis catus* and red foxes *Vulpes vulpes* in our study area (Spencer et al. 2014a,b). Alternatively, as *V. gouldii* is an opportunistic predator that consumes a wide range of vertebrate and invertebrate prey (Cross et al. 2020), it is possible that *V. gouldii* finds and consumes small mammal species in relation to their abundance. Thus, in the long term trapping record for the study area, from 1990, *P. hermannsburgensis* was the most frequently captured small mammal and *S. hirtipes* one of the most scarce (Greenville et al. 2016b). If *V. gouldii* exploits small mammals roughly in relation to their abundance it implies that the per capita risk of predation for each species is similar and could account for the between-species similarity in the GUD results. A larger dietary sample for *V. gouldii* is needed to confirm this.

Another factor that will influence the vulnerability of small mammals to predation from *V. gouldii* is the extent to which this varanid can effectively exploit their burrow odours. The results derived from our second prediction confirm that *V. gouldii* is attracted to small mammal burrow odours but is markedly less interested in burrows that are free of odour. Thus, burrows with no odour were dug into on just seven occasions compared with a total of 450 occasions for burrows containing the odour of a small mammal. On six of the seven occasions when a control burrow was excavated, a burrow containing small mammal odour in the same site cluster was also dug up or investigated. The tracks of *V. gouldii* were recorded at control burrows on 29 occasions, and on most of these the tracks indicated that the varanid had simply walked across the smoothed sand plot without changing stride. By contrast, tracks were recorded at 164 burrows that contained small mammal odour, and at most of these there was evidence that the varanid had broken stride or altered direction to investigate further, even if no subsequent digging occurred. These findings support our second prediction and indicate that *V. gouldii* responds strongly to the burrow odour of small mammals by increasing its exploratory or digging activity. Tracking of prey odours has been documented in other terrestrial vertebrates (Müller-Schwarze 2006), marine predators such as sharks (Tester 1963), and in many insects that follow windborne odour plumes (Cardé 2021), suggesting that olfactory eavesdropping provides benefits for a diverse range of predators.

Despite our results, there is evidence that *V. gouldii* does not respond to the odours of different small mammal species equally. This varanid dug less and showed less investigatory interest (via its tracks) at burrows containing the odour of *N. alexis* than the other species we studied. This result supports part ii of our second prediction that *V. gouldii* should be less attracted to the odours of deep-burrowing species that would be costly to excavate, in contrast to the odours of shallow burrowers such as *P. hermannsburgensis* and *S. youngsoni*. However, *V. gouldii* also dug frequently and showed interest in burrows containing the odour of *R. villosissimus*, which we had not expected. Burrows of the latter species may descend to at least 80 cm and have more than a dozen entry or exit holes that are connected by ~ 20 m of tunnel (Predavec and Dickman 1994). We suggest two possible reasons for the unexpected interest of *V. gouldii* in burrows imbued with the odour of *R. villosissimus*. Firstly, the large size of the rat (120 g) would provide a greater energetic yield once captured than any of the other study species and hence would reward a greater allocation of effort in hunting it. If multiple rats occupy a burrow system, this would additionally maximise the chance that one or more individuals could be captured. Secondly, *R. villosissimus* excavates a second type of burrow that is shallower and simpler than the first. This burrow type is excavated mainly on the sides of dunes (Predavec and Dickman 1994) where most of our artificial burrows were set. Digging *R. villosissimus* from such burrows would likely maximise the energetic returns of *V. gouldii*.

Support for our third prediction, that small mammals would be less mobile and show higher burrow fidelity in the absence than in the presence of *V. gouldii*, was mixed. Rates of movement of small mammals were the same whether *V. gouldii* was present or not, but fidelity to burrows increased in two of the three species that were radio-tracked in the area where *V. gouldii* was removed. Our sample sizes in the tracking experiment were small and rates of movement were highly variable within and between species, so it is possible that our results lacked the power to detect any differences in movements that were present. The rates of movement of both the native rodents were similar to those reported in previous studies (Letnic 2002; Dickman et al. 2010, 2011), as were the greater rates of movement of *S. youngsoni* (Letnic 2002; Haythornthwaite and Dickman 2006). Several authors have interpreted the extensive movements and drifting home ranges of small mammals in arid Australia as responses to continual shifts in the availability of food or other resources across the landscape (e.g., Morton 1978; Read 1984; Dickman et al. 1995; Pavey et al. 2017). However, our radio-tracking took place soon after heavy rains when small mammal populations were at peak abundance and resources could be expected to be reliably present (Dickman et al. 2014; Greenville et al. 2016b). It would be of considerable interest to evaluate whether small mammals exhibit different patterns of movement in response to the presence of *V. gouldii* when conditions are less productive.

Despite the similar rates of movement of our study species across the experimental treatments, both *P. hermannsburgensis* and *S. youngsoni* returned to the same burrows more consistently in the absence than in the presence of *V. gouldii*. Burrows are probably costly to excavate, even in sandy soils, but should repay the return on investment if they provide security and the excavators can return to them on a regular basis. By extension, use of different burrows is likely to be more costly. Additional costs would be incurred if new burrows are continually constructed or, as with *S. youngsoni*, if animals take the risk of using burrows constructed by other fauna that may be hostile to their burrows being shared or usurped. Our results suggest that the presence of *V. gouldii* skews the cost:benefit ratio of burrow use such that the costs of continually moving to different burrows are exceeded by the costs of being detected and depredated by this varanid. In other words, small mammals experience a clear fitness benefit from burrow-hopping if *V. gouldii* is present. If *V. gouldii* is scarce, by contrast, the risk of being discovered in a burrow by the predator is diminished and the benefits of consistent burrow use then result in increased burrow fidelity. Notably, *N. alexis* showed no difference in burrow fidelity between the control and treatment areas. This species digs multi-entrance burrows that descend to 1 m and can evade predators in the open by its rapidity of movement (up to 4.5 m/s: Stanley 1971) and ability to change direction quickly and unpredictably (Watts and Aslin 1981). This species thus may face little risk of injury or death in the presence of *V. gouldii*, even if it is detected, and individuals presumably derive net benefit from making consistent use of their burrows whether the sand goanna is present or absent.

We interpret patterns of burrow usage in small mammals such as *P. hermannsburgensis* and *S. youngsoni* in terms of odour-mediated interactions. On the one hand, high burrow fidelity by these species likely results in accumulations of odours at burrows and their immediate surrounds, providing a strong cue for an olfactory predator such as *V. gouldii* to investigate and excavate them. Low burrow fidelity should reduce these olfactory cues. On the other hand, the observed reduction in burrow fidelity by two of our three study species in the presence of *V. gouldii* implies that they had detected the predator and were responding to it. Although we cannot confirm that olfaction was the modality used, it is the only modality that seems plausible: odour from sources such as goanna faeces is long lasting (> 24 h in our experiments) and clearly recognized by small mammals. It is highly improbable, moreover, that small mammals could reliably use auditory or visual cues to determine the presence of *V. gouldii* owing to the short period of joint overlap in their daily activities. We conclude therefore that olfaction plays a large role in the predator–prey dynamics of this apex reptilian predator and its mammalian prey, and is the most likely driver of the highly unusual and perhaps unique burrow-shifting behaviour that is displayed by many of Australia's small desert mammals. Further experimental work is needed to clarify which infochemicals small mammals use to detect and respond to *V. gouldii* at the landscape level, and whether differences in prey age, health, or experience alter the level of risk that they perceive. Future research could profitably also explore the extent to which infochemicals play a role in shaping other interactions (e.g., diffuse competition Dickman et al. 2020) between small mammals and reptiles elsewhere in arid Australia and desert regions in southern Africa and North America (Pianka and Vitt 2003) where species-rich assemblages of these taxa occur.

## Supplementary Information

Below is the link to the electronic supplementary material.

Supplementary file1 (DOCX 16 KB)

## Data Availability

Not applicable.

## Declarations

### Scientific approval

All components of the research were authorized by the Queensland government's Environmental Protection Agency, Scientific Purposes Permit Numbers WITK07635510 and WITK15192514.

## References

[CR1] Allen ML, Gunther MS, Wilmers CC (2017). The scent of your enemy is my friend? The acquisition of large carnivore scent by a smaller carnivore. J Ethol.

[CR2] Anson JR, Dickman CR (2013). Behavioral responses of native prey to disparate predators: naiveté and predator recognition. Oecologia.

[CR3] Apfelbach R, Blanchard CD, Blanchard RJ, Hayes RA, McGregor IS (2005). The effects of predator odors in mammalian prey species: a review of field and laboratory studies. Neurosci Biobehav Rev.

[CR4] Baker AB, Dickman CR (2018) Secret lives of carnivorous marsupials. CSIRO Publishing, Melbourne. 10.1071/9781486305155

[CR5] Banks PB, Bytheway JP, Carthey AJR, Hughes NK, Price CJ, Glen AS, Dickman CR (2014). Olfaction and predator-prey interactions amongst mammals in Australia. Carnivores of Australia: past, present and future.

[CR6] Banks PB, Daly A, Bytheway JP (2016). Predator odours attract other predators, creating an olfactory web of information. Biol Lett.

[CR7] Bates D, Mächler M, Bolker B, Walker S (2015) Fitting linear mixed-effects models using lme4. J Stat Softw 67:1–48. 10.18637/jss.v067.i01

[CR8] Bedoya-Pérez MA, Smith KL, Kevin RC, Luo JL, Crowther MS, McGregor IS (2019). Parameters that affect fear responses in rodents and how to use them for management. Front Ecol Evol.

[CR9] Bleicher SS, Kotler BP, Shalev O, Dixon A, Embar, K, Brown JS (2018) Divergent behavior amid convergent evolution: a case of four desert rodents learning to respond to known and novel vipers. PLoS ONE 13:e e0200672. 10.1371/journal.pone.0200672 10.1371/journal.pone.0200672 PMC6101362 30125293

[CR10] Bolton J, Moseby K (2004). The activity of sand goannas *Varanus gouldii* and their interaction with reintroduced greater stick-nest rats *Leporillus conditor*. Pac Cons Biol.

[CR11] Brown JS (1988). Patch use as an indicator of habitat preference, predation risk, and competition. Behav Ecol Sociobiol.

[CR12] Burghardt GM, Denny D (1983). Effects of prey movement and prey odor on feeding in garter snakes. Z Tierpsychol.

[CR13] Byrne M, Yeates DK, Joseph L, Kearney M, Bowler J, Williams MAJ, Cooper S, Donnellan SC, Keogh JS, Leys R, Melville J, Murphy DJ, Porch N, Wyrwoll K-H (2008). Birth of a biome: insights into the assembly and maintenance of the Australian arid zone biota. Mol Ecol.

[CR14] Canteras NS, Pavesi E, Carobrez AP (2015). Olfactory instruction for fear: neural system analysis. Front Neurosci.

[CR15] Cardé RT (2021). Navigation along windborne plumes of pheromone and resource-linked odors. Ann Rev Entomol.

[CR16] Carthey AJR, Bytheway JP, Banks PB (2011). Negotiating a noisy, information-rich environment in search of cryptic prey: olfactory predators need patchiness in prey cues. J Anim Ecol.

[CR17] Caspers BA, Schroeder FC, Frank S, Streich WJ, Voigt CC (2009). Odour-based species recognition in two sympatric species of sac-winged bats (*Saccopteryx bilineata*, *S. leptura*): combining chemical analyses, behavioural observations and odour preference tests. Behav Ecol Sociobiol.

[CR18] Chrétien LTS, van der Heide H, Greenberg LO, Giron D, Dicke M, Lucas-Barbosa D (2021). Multiple attack to inflorescences of an annual plant does not interfere with the attraction of parasitoids and pollinators. J Chem Ecol.

[CR19] Churchfield S (1980). Subterranean foraging and burrowing activity of the common shrew. Acta Theriol.

[CR20] Cocke R, Thiessen DD (1986). Chemocommunication among prey and predator species. Anim Learn Behav.

[CR21] Cogger HG (2014). Reptiles & amphibians of Australia.

[CR22] Conover MR (2007). Predator–prey dynamics: the role of olfaction.

[CR23] Cooper T, Liew A, Andrle G, Cafritz E, Dallas H, Niesen T, Slater E, Stockert J, Vold T, Young M, Mendelson J (2019). Latency in problem solving as evidence for learning in varanid and helodermatid lizards, with comments on foraging techniques. Copeia.

[CR24] Cox TE, Murray PJ, Hall GP, Li X (2012). Manipulating resource use by goats with predator fecal odors. Wildl Soc Bull.

[CR25] Cross SL, Craig MD, Tomlinson S, Bateman PW (2020). I don't like crickets, I love them: invertebrates are an important prey source for varanid lizards. J Zool.

[CR26] Dicke M, Sabelis MW (1988). Infochemical terminology: based on cost-benefit analysis rather than origin of compounds?. Funct Ecol.

[CR27] Dickman CR (1992). Predation and habitat shift in the house mouse, *Mus domesticus*. Ecology.

[CR28] Dickman CR (1993). The biology and management of native rodents of the arid zone in New South Wales. New South Wales National Parks and Wildlife Service Species Management Report.

[CR29] Dickman CR, Jones ME, Dickman CR, Archer M (2003). Distributional ecology of dasyurid marsupials. Predators with pouches: the biology of carnivorous marsupials.

[CR30] Dickman CR, Predavec M, Downey FJ (1995). Long-range movements of small mammals in arid Australia: implications for land management. J Arid Env.

[CR31] Dickman CR, Letnic M, Mahon PS (1999). Population dynamics of two species of dragon lizards in arid Australia: the effects of rainfall. Oecologia.

[CR32] Dickman CR, Mahon PS, Masters P, Gibson DF (1999). Long-term dynamics of rodent populations in arid Australia: the influence of rainfall. Wildl Res.

[CR33] Dickman CR, Greenville AC, Beh C-L, Tamayo B, Wardle GM (2010). Social organization and movements of desert rodents during population “booms” and “busts” in central Australia. J Mammal.

[CR34] Dickman CR, Greenville AC, Tamayo B, Wardle GM (2011). Spatial dynamics of small mammals in central Australian desert habitats: the role of drought refugia. J Mammal.

[CR35] Dickman CR, Wardle GM, Foulkes J, de Preu N, Lindenmayer D, Burns E, Thurgate N, Lowe A (2014). Desert complex environments. Biodiversity and environmental change: monitoring, challenges and direction.

[CR36] Dickman CR, Greenville AC, Wardle GM, Bytheway JP (2020). Class conflict: diffuse competition between mammalian and reptilian predators. Diversity.

[CR37] Doody JS, Soennichsen KF, James H, McHenry C, Clulow S (2020). Ecosystem engineering by deep-nesting monitor lizards. Ecology.

[CR38] Downey FJ, Dickman CR (1993) Macro and microhabitat relationships among lizards of sandridge desert in central Australia. In: Lunney D, Ayers D (eds) Australian Herpetology: a diverse discipline. Royal Zoological Society of New South Wales, Sydney, pp 133–138. 10.7882/RZSNSW.1993.020

[CR39] Fardell LL, Pavey CR, Dickman CR (2020). Fear and stressing in predator-prey ecology: considering the twin stressors of predators and people on mammals. PeerJ.

[CR40] Fardell LL, Bedoya-Pérez MA, Dickman CR, Crowther MS, Pavey CR, Narayan EJ (2021). Are physiological and behavioural responses to stressors displayed concordantly by wild urban rodents?. Science of Nat.

[CR41] Fendt M, Parsons MH, Apfelbach R, Carthey AJR, Dickman CR, Endres T, Frank ASK, Heinz DE, Jones ME, Kiyokawa Y, Kreutzmann JC, Roelofs K, Schneider M, Sulger J, Wotjak CT, Blumstein DT (2020). Context and trade-offs characterize real-world threat detection systems: a review and comprehensive framework to improve research practice and resolve the translational crisis. Neurosci Biobehav Rev.

[CR42] Fenn MG, Macdonald DW (1995). Use of middens by red foxes: risk reverses rhythms of rats. J Mammal.

[CR43] Fisher DO, Dickman CR (1993). Diets of insectivorous marsupials in arid Australia: selection for prey type, size or hardness?. J Arid Env.

[CR44] Gable TD, Windels SK, Bruggink JG, Homkes AT (2016). Where and how wolves (*Canis lupus*) kill beavers (*Castor canadensis*). PLoS ONE.

[CR45] Garrett CM, Card WC (1993). Chemical discrimination of prey by naive neonate Gould's monitors *Varanus gouldii*. J Chem Ecol.

[CR46] Garrett CM, Boyer DM, Card WC, Roberts DT, Murphy JB, Chiszar D (1996). Comparison of chemosensory behavior and prey trail-following in the varanoid lizards *Varanus gouldii* and *Heloderma suspectum*. Zoo Biol.

[CR47] Garvey PM, Glen AS, Clout MN, Wyse SV, Nichols M, Pech RP (2017). Exploiting interspecific olfactory communication to monitor predators. Ecol Appl.

[CR48] Gordon CE, Dickman CR, Thompson MB (2010). Partitioning of temporal activity among desert lizards in relation to prey availability and temperature. Austral Ecol.

[CR49] Grau C, Teruel E, Leclercq J, Pageat P (2019) House mouse (*Mus musculus*) avoidance of olfactory cues from ferrets and other mammalian and reptilian predators: preliminary results. In: Buesching CD (ed) Chemical signals in vertebrates 14. Springer Nature, Switzerland, pp 165–181. 10.1007/978-3-030-17616-7_13

[CR50] Greenville AC, Dickman CR, Wardle GM, Letnic M (2009). The fire history of an arid grassland: the influence of antecedent rainfall and ENSO. Int J Wildland Fire.

[CR51] Greenville AC, Wardle GM, Dickman CR (2012). Extreme climatic events drive mammal irruptions: regression analysis of 100-year trends in desert rainfall and temperature. Ecol Evol.

[CR52] Greenville AC, Wardle GM, Dickman CR (2013). Extreme rainfall events predict irruptions of rat plagues in central Australia. Austral Ecol.

[CR53] Greenville AC, Wardle GM, Nguyen V, Dickman CR (2016). Spatial and temporal synchrony in reptile population dynamics in variable environments. Oecologia.

[CR54] Greenville AC, Wardle GM, Nguyen V, Dickman CR (2016). Population dynamics of desert mammals: similarities and contrasts within a multispecies assemblage. Ecosphere.

[CR55] Hartig F (2021) DHARMa: Residual diagnostics for hierarchical (multi-level / mixed) regression models. R package version 0.4.0. https://CRAN.R-project.org/package=DHARMa

[CR56] Haythornthwaite AS, Dickman CR (2006). Long-distance movements by a small carnivorous marsupial: how *Sminthopsis youngsoni* (Marsupialia: Dasyuridae) uses habitat in an Australian sandridge desert. J Zool.

[CR57] Hughes NK, Price CJ, Banks PB (2010). Predators are attracted to the olfactory signals of prey. PLoS ONE.

[CR58] Ibáñez-Álamo JD, Magrath RD, Oteyza JC, Chalfoun AD, Haff TM, Schmidt KA, Thomson RL, Martin TE (2015). Nest predation research: recent findings and future perspectives. J Ornithol.

[CR59] Jones ME, Apfelbach R, Banks PB, Cameron EZ, Dickman CR, Frank A, McLean S, McGregor IS, Müller-Schwarze D, Parsons MH, Sparrow E, Blumstein DT (2016) A nose for death: integrating trophic and informational networks for conservation and management. Frontiers Ecol Evol 4:Article 124. 10.3389/fevo.2016.00124

[CR60] Kaufman JD, Burghardt GM, Phillips JA (1996). Sensory cues and foraging decisions in a large carnivorous lizard, *Varanus albigularis*. Anim Behav.

[CR61] Kenward RE (2001). A manual for wildlife radio tagging.

[CR62] Kotler BP, Brown JS, Hasson O (1991). Owl predation on gerbils: the role of body size, illumination, and habitat structure on rates of predation. Ecology.

[CR63] Kovacs EK, Crowther MS, Webb JK, Dickman CR (2012). Population and behavioural responses of native prey to alien predation. Oecologia.

[CR64] Lenth RV (2021) emmeans: estimated marginal means, aka least-squares means. R package version 1.5.5–1. https://CRAN.R-project.org/package=emmeans

[CR65] Letnic M (2002). Long distance movements and the use of fire mosaics by small mammals in the Simpson Desert, central Australia. Aust Mammal.

[CR66] Lewis ND, Breckels MN, Steinke M, Codling EA (2013). Role of infochemical mediated zooplankton grazing in a phytoplankton competition model. Ecol Complex.

[CR67] Losos JB, Greene HW (1988). Ecological and evolutionary implications of diet in monitor lizards. Biol J Linn Soc.

[CR68] MacArthur RH, Pianka ER (1966). On optimal use of a patchy environment. Am Nat.

[CR69] Magurran AE (2004). Measuring biological diversity.

[CR70] Manrod JD, Hartdegen R, Burghardt GM (2008). Rapid solving of a problem apparatus by juvenile black-throated monitor lizards (*Varanus albigularis albigularis*). Anim Cogn.

[CR71] Morton SR (1978). An ecological study of *Sminthopsis crassicaudata* (Marsupialia: Dasyuridae) II. Behaviour and social organization. Aust Wildl Res.

[CR72] Moseby K, Nano T, Southgate R (2009) Tales in the sand: a guide to identifying Australian arid zone fauna using spoor and other signs. Tracking Australia, Adelaide

[CR73] Müller C, Caspers BA, Gadau J, Kaiser S (2020). The power of infochemicals in mediating individualized niches. Trends Ecol Evol.

[CR74] Müller-Schwarze D (2006). Chemical ecology of vertebrates.

[CR75] Murray BR, Dickman CR (1994). Granivory and microhabitat use in Australian desert rodents: are seeds important?. Oecologia.

[CR76] Murray BR, Dickman CR, Watts CHS, Morton SR (1999). The dietary ecology of Australian desert rodents. Wildl Res.

[CR77] Newman C and Buesching CD (2019) Detecting the smell of disease and injury: scoping evolutionary and ecological implications. In: Buesching CD (ed) Chemical signals in vertebrates 14. Springer Nature, Switzerland, pp 238–250. 10.1007/978-3-030-17616-7_17

[CR78] Norbury GL, Price CJ, Latham MC, Brown SJ, Latham ADM, Brownstein GE, Ricardo HC, McArthur NJ, Banks PB (2021) Misinformation tactics protect rare birds from problem predators. Sci Adv 7:eabe4164. 10.1126/sciadv.abe4164 10.1126/sciadv.abe4164 PMC7946364 33692107

[CR79] Parsons MH, Blumstein DT (2010). Familiarity breeds contempt: kangaroos persistently avoid areas with experimentally deployed dingo scents. PLoS ONE.

[CR80] Parsons MH, Blumstein DT (2010). Feeling vulnerable? Indirect risk cues differently influence how two marsupials respond to novel dingo urine. Ethology.

[CR81] Parsons MH, Apfelbach R, Banks PB, Cameron EZ, Dickman CR, Frank ASK, Jones ME, McGregor IS, McLean S, Müller-Schwarze D, Sparrow EE, Blumstein DT (2018). Biologically meaningful scents: a framework for understanding predator–prey research across disciplines. Biol Rev.

[CR82] Pavey CR, Addison J, Brandle R, Dickman CR, McDonald PJ, Moseby KE, Young LI (2017). The role of refuges in the persistence of Australian dryland mammals. Biol Rev.

[CR83] Pianka ER (1970). Notes on the biology of *Varanus gouldi flavirufus*. WA Nat.

[CR84] Pianka ER (1994). Comparative ecology of *Varanus* in the Great Victoria Desert. Aust J Ecol.

[CR85] Pianka ER, Vitt LJ (2003). Lizards: windows to the evolution of diversity.

[CR86] Pinheiro J, Bates D, DebRoy S, Sarkar D (2021) nlme: linear and nonlinear mixed effects models. R package version 3.1–152. https://CRAN.R-project.org/package=nlme

[CR87] Predavec M, Dickman CR (1994). Population dynamics and habitat use of the long-haired rat (*Rattus villosissimus*) in south-western Queensland. Wildl Res.

[CR88] Price CJ, Banks PB (2012). Exploiting olfactory learning in alien rats to protect birds' eggs. PNAS.

[CR89] R Core Team (2021) R: A language and environment for statistical computing. R Foundation for Statistical Computing, Vienna, Austria. URL https://www.R-project.org/

[CR90] Radford IJ, Woolley L-A, Dickman CR, Corey B, Trembath D, Fairman R (2020). Invasive anuran driven trophic cascade: an alternative hypothesis for recent critical weight range mammal collapses across northern Australia. Biol Inv.

[CR91] Read DG (1984). Movements and home ranges of three sympatric dasyurids, *Sminthopsis crassicaudata*, *Planigale gilesi* and *P. tenuirostris* (Marsupialia), in semiarid western New South Wales. Aust Wildl Res.

[CR92] Salo P, Korpimäki E, Banks PB, Nordström M, Dickman CR (2007). Alien predators are more dangerous than native predators to prey populations. Proc R Soc B.

[CR93] Savidge JA (1987). Extinction of an island forest avifauna by an introduced snake. Ecology.

[CR94] Scogings PF, Demmer S, Hattas D (2021). Spinescence and total phenolic content do not influence diet preference of a critically endangered megaherbivore, but the mix of compounds does. J Chem Ecol.

[CR95] Sih A, Bolnick DI, Luttbeg B, Orrock JL, Peacor SD, Pintor LM, Preisser E, Rehage JS, Vonesh JR (2010). Predator–prey naïveté, antipredator behavior, and the ecology of predator invasions. Oikos.

[CR96] Spencer EE, Crowther MS, Dickman CR (2014). Risky business: do native rodents use habitat and odor cues to manage predation risk in Australian deserts?. PLoS ONE.

[CR97] Spencer EE, Crowther MS, Dickman CR (2014). Diet and prey selectivity of three species of sympatric mammalian predators in central Australia. J Mammal.

[CR98] Stanley M (1971). An ethogram of the hopping mouse, *Notomys alexis*. Z Tierpsychol.

[CR99] Stobo-Wilson AM, Murphy BP, Legge SM, Chapple DG (2021). Reptiles as food: predation of Australian reptiles by introduced red foxes compounds and complements predation by cats. Wildl Res.

[CR100] Stoffel MA, Caspers BA, Forcade J, Giannakara A, Baier M, Eberhart-Phillips L, Müller C, Hoffman JI (2015). Chemical fingerprints encode mother-offspring similarity, colony membership, relatedness, and genetic quality in fur seals. Proc Nat Acad Sci USA.

[CR101] Sundaram M, Higdon AE, Wood KV, Bonham CC, Swihart RK (2020). Mechanisms underlying detection of seed dormancy by a scatter-hoarding rodent. Int Zool.

[CR102] Sutherland DR, Bryant GL, Glen AS, Dickman CR (2014). Reptilian predators: the forgotten majority?. Carnivores of Australia: past, present and future.

[CR103] Sutherland DR, Glen AS, de Tores PJ (2011). Could controlling mammalian carnivores lead to mesopredator release of carnivorous reptiles?. Proc R Soc B.

[CR104] Takahashi LK (2014). Olfactory systems and neural circuits that modulate predator odor fear. Front Behav Neurosci.

[CR105] Taraborelli P, Borruel N, Mangeaud A (2009). Ability of murid rodents to find buried seeds in the Monte Desert. Ethology.

[CR106] Tester AL (1963). The role of olfaction in shark predation. Pac Sci.

[CR107] Thompson GG (1995). Foraging patterns and behaviours, body postures and movement speed for goannas, *Varanus gouldii* (Reptilia: Varanidae), in a semi-urban environment. J Roy Soc WA.

[CR108] Van Dyck S, Strahan R (2008). The mammals of Australia.

[CR109] Verhoeven EM, Murray BR, Dickman CR, Wardle GM, Greenville AC (2020). Fire and rain are one: extreme rainfall events predict wildfire extent in an arid grassland. Int J Wildland Fire.

[CR110] Vos M, Vet LEM, Wäckers FL, Middelburg JJ, van der Putten WH, Mooij WM, Heip CHR, van Donk E (2006). Infochemicals structure marine, terrestrial and freshwater food webs: implications for ecological informatics. Ecol Info.

[CR111] Wardle GM, Greenville AC, Frank ASK, Tischler M, Emery NJ, Dickman CR (2015). Ecosystem risk assessment of Georgina gidgee woodlands in central Australia. Austral Ecol.

[CR112] Watts CHS, Aslin HJ (1981). The rodents of Australia.

[CR113] Webb JK, Shine R (1992). To find an ant: trail-following in Australian blindsnakes. Anim Behav.

[CR114] White GC, Garrott RA (1990). Analysis of wildlife radio-tracking data.

[CR115] Woinarski JCZ, Murphy BP, Palmer R, Legge SM, Dickman CR, Doherty TS, Edwards G, Nankivell A, Read JL, Stokeld D (2018). How many reptiles are killed by cats in Australia?. Wildl Res.

[CR116] Zuur AF, Ieno EN, Walker NJ, Saveliev AA, Smith GM (2009). Mixed effects models and extensions in ecology with R.

