# Supplementary material for: Odour-mediated Interactions Between an Apex Reptilian Predator and its Mammalian Prey
Source: J Chem Ecol. 2022 Mar 1;48(4):401–15. doi: 10.1007/s10886-022-01350-w (PMC9079038; doi:10.1007/s10886-022-01350-w)
Supplement: Supplementary file 1 — Supplementary file1 (DOCX 16 KB) [file 10886_2022_1350_MOESM1_ESM.docx]

**Online Resource 1**

**Journal of Chemical Ecology**

**Odour-mediated Interactions Between an Apex Reptilian Predator and its Mammalian Prey**

Christopher R. Dickman*, Loren L. Fardell, Nicole Hills

*Desert Ecology Research Group, School of Life and Environmental Sciences, The University of Sydney, NSW 2006, Australia*

*Corresponding author: chris.dickman@sydney.edu.au

**Diet of the sand goanna *Varanus gouldii***

Our goal in assessing the diet of *V. gouldii* was to quantify the extent to which this reptilian predator consumes small mammals. We captured *V. gouldii* during a long-term live trapping program in the study area (Dickman et al. 2014; Greenville et al. 2016) or by hand noosing following opportunistic sightings. Except when needed for odour experiments (see below), any faeces that were produced while animals were in traps or during handling were placed into labelled paper or zip-lock bags, then dried and stored. Mammalian hairs were later extracted and identified to species using the diagnostic methods (hair cross sections and cuticular scale patterns) and reference keys of Brunner et al. (2002). Hair analysis was completed by Georgeanna Story (Scats About Pty, Majors Creek, NSW) and the authors.

We report the frequency of occurrence of the mammalian study species in faecal samples of *V. gouldii* as the percentage of the overall sample size.

Invertebrates dominated the diet of *V. gouldii*, occurring in 172 of 178 faecal samples (96.6%). Small mammals occurred in 66 faecal samples (37%), with the house mouse *Mus musculus* represented in 30 samples (16.9%). The sandy inland mouse *Pseudomys hermannsburgensis* occurred in 13 faecal samples (7.3%), spinifex hopping-mouse *N. alexis* and long-haired rat *R. villosissimus* in five samples each (2.8%) and the lesser hairy-footed dunnart *S. youngsoni* in eight samples (4.5%). Two rarely captured native rodents, the desert mouse *Pseudomys desertor* and Forrest's mouse *Leggadina forresti*, occurred in the remaining five faecal samples. Although small numbers of hairy-footed dunnarts *Sminthopsis hirtipes* were captured during long-term monitoring in the study area, no hairs of this species were detected in faecal samples of *V. gouldii*.

**References**

Brunner H, Triggs B, Ecobyte Pty Ltd (2002) Hair ID: an interactive tool for identifying Australian mammalian hair. CSIRO Publishing, Melbourne

Dickman CR, Wardle GM, Foulkes J, de Preu, N (2014) Desert complex environments. In Lindenmayer D, Burns E, Thurgate N, Lowe A (eds) Biodiversity and environmental change: monitoring, challenges and direction. CSIRO Publishing, Melbourne, pp 379–438

Greenville AC, Wardle GM, Nguyen V, Dickman CR (2016) Spatial and temporal synchrony in reptile population dynamics in variable environments. Oecologia 182:475$-$485. <https://doi.org/10.1007/s00442-016-3672-8>
